# Impact of admission hyperglycemia on short and long-term prognosis in acute myocardial infarction: MINOCA versus MIOCA

**DOI:** 10.1186/s12933-021-01384-6

**Published:** 2021-09-24

**Authors:** Pasquale Paolisso, Alberto Foà, Luca Bergamaschi, Francesco Angeli, Michele Fabrizio, Francesco Donati, Sebastiano Toniolo, Chiara Chiti, Andrea Rinaldi, Andrea Stefanizzi, Matteo Armillotta, Angelo Sansonetti, Ilenia Magnani, Gianmarco Iannopollo, Paola Rucci, Gianni Casella, Nazzareno Galiè, Carmine Pizzi

**Affiliations:** 1 Department of Experimental, Diagnostic and Specialty Medicine-DIMES, IRCCS Policlinico S. Orsola - Malpighi, University of Bologna, Via Giuseppe Massarenti 9, 40138 Bologna, Italy; 2 Unit of Cardiology, Maggiore Hospital, Bologna, Italy; 3 Division of Hygiene and Biostatistics, Department of Biomedical and Neuromotor Sciences, Alma Mater Studiorum, University of Bologna, Bologna, Italy

**Keywords:** MINOCA, MIOCA, Stress-hyperglycemia, Acute myocardial infarction, Short-term prognosis, Long-term prognosis

## Abstract

**Background:**

The prognostic role of hyperglycemia in patients with myocardial infarction and obstructive coronary arteries (MIOCA) is acknowledged, while data on non-obstructive coronary arteries (MINOCA) are still lacking. Recently, we demonstrated that admission stress-hyperglycemia (aHGL) was associated with a larger infarct size and inflammatory response in MIOCA, while no differences were observed in MINOCA. We aim to investigate the impact of aHGL on short and long-term outcomes in MIOCA and MINOCA patients.

**Methods:**

Multicenter, population-based, cohort study of the prospective registry, designed to evaluate the prognostic information of patients admitted with acute myocardial infarction to S. Orsola-Malpighi and Maggiore Hospitals of Bologna metropolitan area. Among 2704 patients enrolled from 2016 to 2020, 2431 patients were classified according to the presence of aHGL (defined as admission glucose level ≥ 140 mg/dL) and AMI phenotype (MIOCA/MINOCA): no-aHGL (n = 1321), aHGL (n = 877) in MIOCA and no-aHGL (n = 195), aHGL (n = 38) in MINOCA. Short-term outcomes included in-hospital death and arrhythmias. Long-term outcomes were all-cause and cardiovascular mortality.

**Results:**

aHGL was associated with a higher in-hospital arrhythmic burden in MINOCA and MIOCA, with increased in-hospital mortality only in MIOCA. After adjusting for age, gender, hypertension, Killip class and AMI phenotypes, aHGL predicted higher in-hospital mortality in non-diabetic (HR = 4.2; 95% CI 1.9–9.5, p = 0.001) and diabetic patients (HR = 3.5, 95% CI 1.5–8.2, p = 0.003). During long-term follow-up, aHGL was associated with 2-fold increased mortality in MIOCA and a 4-fold increase in MINOCA (p = 0.032 and p = 0.016). Kaplan Meier 3-year survival of non-hyperglycemic patients was greater than in aHGL patients for both groups. No differences in survival were found between hyperglycemic MIOCA and MINOCA patients. After adjusting for age, gender, hypertension, smoking, LVEF, STEMI/NSTEMI and AMI phenotypes (MIOCA/MINOCA), aHGL predicted higher long-term mortality.

**Conclusions:**

aHGL was identified as a strong predictor of adverse short- and long-term outcomes in both MIOCA and MINOCA, regardless of diabetes. aHGL should be considered a high-risk prognostic marker in all AMI patients, independently of the underlying coronary anatomy.

*Trial registration* data were part of the ongoing observational study AMIPE: Acute Myocardial Infarction, Prognostic and Therapeutic Evaluation. ClinicalTrials.gov Identifier: NCT03883711.

**Supplementary Information:**

The online version contains supplementary material available at 10.1186/s12933-021-01384-6.

## Introduction

Admission stress hyperglycemia (aHGL) frequently occurs in patients hospitalized for acute myocardial infarction (AMI) in both diabetic and non-diabetic patients [1–3]. The prevalence of aHGL ranges from 25 to 50% depending on the hyperglycemia definition cut-off adopted [1, 4]. The American Heart Association and the Endocrine Society Clinical Guidelines defined stress hyperglycemia as a random plasma glucose level above 140 mg/dL at any given time for both diabetic and non-diabetic hospitalized patients [2, 3, 5]. To date, there is growing evidence that aHGL negatively affects short and long-term outcomes in AMI patients, independently of a concomitant diabetic status [6–9]. Nowadays, it is well known that type 2 diabetes mellitus (T2DM) is a common comorbidity in patients with cardiovascular diseases [10]. Specifically, T2DM is detected in more than 20% of patients admitted for suspected AMI, conferring a 2-fold in-hospital mortality increase and a higher risk of major adverse cardiovascular events (MACE) during follow-up [11–13]. Recent studies have shown that among AMI with Non-Obstructive Coronary Artery disease (MINOCA) patients, T2DM is less common but still an independent predictor of all‐cause mortality over time [14, 15].

In the last years, our knowledge regarding the natural history of MINOCA has changed from an initial favorable prognosis to a substantial risk of recurrent MACE during follow-up [16]. MINOCA represents an extremely heterogeneous clinical entity, as multiple pathophysiological mechanisms may result in variable outcomes [17]. The available prognostic indicators derive mainly from invasive studies, while solid evidence on the impact of clinical risk factors is currently lacking [18, 19]. So far, the scientific community has focused on the role of classic cardiovascular risk factors among MINOCA scenarios, identifying T2DM as a predictor of MACE and mortality [14, 15].

Based on current evidence, in patients with myocardial infarction with obstructive coronary arteries (MIOCA), admission hyperglycemia is associated with larger myocardial necrosis and adverse outcomes, while among MINOCA, which represents a population with a different etiology and a trivial infarct size, the prognostic impact of stress hyperglycemia is still unexplored [20, 21]. Therefore, with the present study we sought to investigate the prognostic role of hyperglycemia on short and long-term prognosis in patients with MINOCA versus MIOCA.

## Methods

### Study design and population

This study enrolled patients from the AMIPE Registry, a prospective, observational cohort study designed to evaluate the prognostic information of patients admitted with AMI to S. Orsola-Malpighi and Maggiore Hospitals of Bologna metropolitan area. From January 2016 to September 2020, all consecutive patients admitted with AMI (both STEMI and NSTEMI) who performed coronary angiography (CAG) were included in the study. STEMI and NSTEMI diagnosis and time for the coronary angiography were managed according to the current guidelines [22, 23]. Based on the presence and extent of stenoses, patients were classified into MIOCA (stenosis ≥ 50% of the lumen diameter in at least one coronary artery) and MINOCA according to the ESC MINOCA Position Paper criteria [24]. Specifically, all patients with non-obstructive coronary arteries underwent pulmonary and vascular computed tomography and contrast cardiac magnetic resonance to exclude non-ischemic troponin elevation causes [25]. Patients with unavailable admission glycemia, type 1 diabetes and concomitant glucocorticoid therapy at the time of admission were excluded from the study. Further exclusion criteria encompassed severe valvular heart diseases, major acute bleeding, severe hepatic impairment, concomitant neoplastic diseases, follow-up data shorter than 6 months. Data were collected as part of an approved protocol regarding the ongoing observational study “AMIPE: Acute Myocardial Infarction, Prognostic and Therapeutic Evaluation” (ClinicalTrials.gov Identifier: NCT03883711). The present study was conducted according to the Declaration of Helsinki; all patients were informed about their participation in the registry and provided informed consent for the anonymous publication of scientific data.

### Follow up and outcomes

Patient were followed over time with outpatient visits and telephone contacts using a standard questionnaire. Short-term outcomes included length of hospital stay, in-hospital death (IHD), and in-hospital arrhythmias. Long-term outcomes were all-cause mortality, cardiovascular mortality, re-hospitalization for MI, heart failure, stroke, MACE, and major adverse events (MAE). Pre-specified endpoints were all-cause mortality, cardiovascular deaths, arrhythmias, myocardial infarction, heart failure, ischemic stroke, and MACE. Definition of clinical endpoints is reported in Additional file 1.

### Statistical analysis

Continuous variables were summarized using mean and standard deviation or median and interquartile range as appropriate. Categorical variables were summarized using absolute and relative frequencies and compared between groups using the Chi-square test. To identify patients’ subgroups with different mortality rates, we carried out a classification tree analysis with split-sample validation in two random training and testing subgroups, including 50% of the study population. MINOCA/MIOCA was forced as the first classification variable, and diabetes and hyperglycemia were used as the independent variables. Hyperglycemia (≥ 140 mg/dL) proved to be the most important variable discriminating patients with significantly different mortality rates and was therefore used to subdivide patients into hyperglycemic (aHGL) and non-hyperglycemic (no-aHGL). Survival was estimated using Kaplan–Meier curves and compared among the study groups using the log-rank test. Cox proportional hazard regression was used to determine the independent predictors of mortality in the overall sample. The proportional risks assumption underlying Cox regression models was tested using Schoenfeld residuals. Clinically relevant variables selected a priori were included in the model with a forward stepwise procedure. The probability for entry and removal from the model were respectively p = 0.05 and p = 0.10. Simple and multiple logistic regression models were used to predict and compare the clinical outcomes (death, stroke, HF, re-AMI, MACE and MAE) among the study groups. Predicted probability of death across continuous admission blood glucose levels values was calculated based on the Cox proportional hazard regression model where the covariate admission glycemia was included as restricted cubic spline in a cubic polynomial regression model. All analyses were performed using the Statistical Package for Social Sciences, version 25.0 (SPSS, PC version, Chicago, IL, USA) and R version 3.5.2 (R Foundation for Statistical Computing, Vienna, Austria).

## Results

### Sample overview

As shown in the study flowchart (Additional file 1: Figure S1), our final study population consisted of 2431 patients hospitalized for AMI (both STEMI and NSTEMI) who underwent coronary angiography and consequently classified as MIOCA (n = 2198) and MINOCA (n = 233). The median times from first EKG to diagnostic angiography for MIOCA and MINOCA patients were 1 [IQR 0.7–1.8] and 0.8 [IQR 0.5–2] hours for STEMI and 26 [IQR 11.6–38] and 28 [IQR 12–39] hours for NSTEMI, respectively. The underlying etiopathological causes and the angiographic results are reported in Additional file 1: Table S1. Admission hyperglycemia was observed in 37.6% of cases, more frequently among MIOCA than MINOCA (39.9% vs 16.3%, p < 0.001). The distribution of glycemic levels in each subgroup is shown in Additional file 1: Figure S2. Based on admission glucose values, MIOCA and MINOCA cohorts were subdivided into aHGL and non-aHGL groups, and all the subsequent analyses were performed accordingly. Clinical characteristics and laboratory/instrumental findings of the four subgroups are reported in Table 1.

**Table 1:** Demographic characteristics, comorbidities and in-hospital admission findings of MIOCA and MINOCA ACS patients, according to admission hyperglycemia

	MIOCA N = 2198			MINOCA N = 233			HGL MIOCA vs MINOCA
	no-aHGL N = 1321	aHGL N = 877	p-value	no-aHGL N = 195	aHGL N = 38	p-value	p-value
Age, years, median [IQR]	69 [58–78]	73 [63–81]	< 0.001	68 [53–77]	74 [67–81]	< 0.001	ns
Gender Female, n (%)	341 (25.8)	262 (30)	0.037	126 (64.6)	26 (68.4)	ns	< 0.001
BMI kg/m^2^, median [IQR]	26.2 [24–29.3]	27 [24.3–30.1]	0.001	25.7 [22.5–28.5]	26 [23.1–29.3]	0.001	ns
Cardiovascular risk factors							
Current/past smoking, n (%)	812 (61.5)	482 (55)	0.002	87 (44.6)	13 (34.2)	ns	0.0012
Hypertension, n (%)	855 (67)	654 (74.6)	< 0.001	126 (64.6)	29 (76.3)	ns	ns
Dyslipidemia, n (%)	814 (61.6)	531 (60.5)	ns	123 (63.1)	19 (50)	ns	ns
Type-2 diabetes, n (%)	118 (9)	458 (52.2)	< 0.001	13 (6.7)	15 (39.5)	< 0.001	ns
De novo Type-2 diabetes, n (%)	7 (0.6)	31 (7)	< 0.001	2 (1.1)	3 (13)	< 0.001	ns
Medical history							
Previous AMI, n (%)	268 (20.4)	216 (24.7)	0.016	18 (10)	2 (5.7)	ns	0.001
Previous stroke, n (%)	72 (5.5)	72 (8.2)	0.01	10 (5.1)	2 (5.3)	ns	ns
COPD, n (%)	138 (10.5)	115 (13.1)	ns	21 (10.8)	5 (13.2)	ns	ns
CKD, n (%)	298 (23.1)	334 (39)	< 0.001	36 (18.8)	14 (37.8)	0.011	ns
PAD, n (%)	81 (6.1)	93 (10.6)	< 0.001	4 (2.1)	2 (5.3)	ns	ns
Hospital admission							
Angina, n (%)	1008 (76.5)	589 (68)	< 0.001	133 (68.6)	13 (34.2)	< 0.001	< 0.001
HR, median [IQR]	72 [61–86]	81 [69–99]	< 0.001	72 [63–88]	97 [72–118]	< 0.001	0.019
SBP, median [IQR]	140 [120–160]	140 [120–160]	ns	140 [120–155]	140 [120–160]	ns	ns
DBP, median [IQR]	80 [70–90]	80 [70–90]	ns	80 [70–90]	80 [70–85]	ns	ns
Atrial fibrillation, n (%)	86 (6.6)	97 (11.1)	< 0.001	14 (7.2)	12 (31.6)	< 0.001	< 0.001
STEMI, n (%)	571 (43.2)	416 (47.4)	ns	22 (11.3)	5 (13.2)	ns	< 0.001
PCI total, n (%)	1113 (84.3)	751 (85.6)	0.38	/	/		/
PCI/NSTEMI, n (%)	601/750 (80.1)	366/461 (79.4)	0.76	/	/		/
Killip class III/IV, n (%)	31 (2.4)	116 (13.3)	< 0.001	3 (1.6)	4 (10.8)	0.003	ns
LVEDV, mL median [IQR]	100 [83–121]	108 [85–135]	0.004	90 [74–107]	82 [70–122]	ns	0.023
LV EF %, median [IQR]	55 [45–60]	46 [40–56]	< 0.001	60 [53–62]	60 [50–62]	ns	< 0.001
Peak hs Troponin I ng/L, median [IQR]	2751 [545–17182]	6334 [999–34431]	< 0.001	461 [109–1691]	370 [136–777]	ns	< 0.001
aBGL level mg/dL, median [IQR]	110 [99–122]	180 [155–234]	< 0.001	104 [93–116]	187 [157–228]	< 0.001	ns
Creatinine, median [IQR]	0.9 [0.8–1.1]	1 [0.9–1.3]	< 0.001	0.8 [0.7–1]	1 [0.77–1.2]	0.017	0.038
eGFR_CKDEPI, median [IQR]	78 [61–91]	67 [49–84]	< 0.001	85 [63–98]	67 [54–79]	< 0.001	ns
BNP pg/mL, median [IQR]	367 [154–723]	514 [192–957]	0.033	159 [72–357]	707 [354–1370]	< 0.008	ns
GRACE score, median [IQR]	136 [115–159]	153 [129–180]	< 0.001	122 [99–144]	154 [128–181]	< 0.001	ns

Continuous variables are presented as median (IQR) while categorical ones as n (%)

*no-aHGL* admission normal glucose level, *aHGL* admission high glucose level, *BMI* body max index, *AMI* acute myocardial infarction, *COPD* chronic obstructive pulmonary disease, *CKD* chronic kidney disease, *PAD* peripheral artery disease, *HR* heart rate, *SBP* systolic blood pressure, *DBP* diastolic blood pressure, *STEMI* ST-segment elevation myocardial infarction, *NSTEMI* non-ST segment elevation myocardial infarction, *PCI* percutaneous coronary intervention, *LVEDV* left-ventricular-end-diastolic-volume, *LVEF* left ventricular ejection fraction, *aBGL* admission blood glucose level

### Hyperglycemic vs non-hyperglycemic patients

Among both MIOCA and MINOCA cohorts, aHGL patients were older, more frequently overweight and with a higher prevalence of chronic kidney disease (Table 1). Hypertension was more prevalent among hyperglycemic MIOCA cases, while no statistically significant differences were detected in MINOCA cases. As expected, a history of T2DM, as well as a newly diagnosed DM, were more frequently observed among hyperglycemic subjects, with no differences in relation to the coronary anatomy. Regarding the clinical presentation, MIOCA and MINOCA hyperglycemic patients less frequently exhibited typical angina, while a higher heart rate and Killip class and a greater prevalence of atrial fibrillation were more often observed. Paralleling such worse clinical and hemodynamic profile, aHGL was associated with a higher GRACE score in both MIOCA and MINOCA cohorts. When comparing the two hyperglycemic subgroups, aHGL MIOCA patients were characterized by higher troponin levels, greater LV end-diastolic volumes (LVEDVs) and a depressed LV function, all markers of larger infarct size (Table 1). Admission and discharge therapy, as well as in-hospital glucose-lowering strategies, are provided in Additional file 1: Table S2.

### MINOCA vs MIOCA outcomes

Overall, 52 patients—50 cases with MIOCA and 2 MINOCA subjects—died during hospitalization, all due to cardiovascular causes. In the MIOCA group, in-hospital mortality was significantly higher in aHGL patients (4.6% vs 0.8%, p < 0.001). Notably, no deaths were observed in normoglycemic diabetic patients. In both MIOCA and MINOCA cohorts, hyperglycemic patients exhibited a greater arrhythmogenic burden during hospitalization—ventricular arrhythmias and atrial fibrillation—when compared to normoglycemic cases (Table 2). Interestingly, hyperglycemic MIOCA patients required mechanical circulatory support with intra-aortic balloon pump 4-times more often than normoglycemic ones (p < 0.001). Additionally, only aHGL MIOCA patients had a longer hospital stay compared to the other subgroups. No significant differences were noticed for short-term outcomes between hyperglycemic MIOCA and MINOCA patients (Table 2).

**Table 2:** Short and long-term outcomes of MIOCA and MINOCA ACS patients, according to admission hyperglycemia

	MIOCA N = 2198			MINOCA N = 233			HGL MIOCA vs MINOCA
	no-aHGL N = 1321	aHGL N = 877	p-value	no-aHGL N = 195	aHGL N = 38	p-value	p-value
Short-term outcomes							
In-hospital death, n (%)	10 (0.8)	40 (4.6)	< 0.001	1 (0.5)	1 (2.6)	ns	ns
With T2DM	0 (0)	20 (4.4)	0.020	–	–	–	
Intra-hospital arrhythmias, n (%)	59 (4.5)	84 (9.7)	< 0.001	5 (2.6)	5 (13.5)	0.003	ns
Atrial fibrillation, n (%)	39 (3)	52 (6)		2 (1)	3 (7.9)		
Ventricular arrhythmias, n (%)	20 (1.5)	32 (3.6)		3 (1.5)	2 (5.7)		
IABP, n (%)	12 (0.9)	34 (3.9)	< 0.001	–	–	–	
Hospital length of stay days, median [IQR]	5 [4–7]	6 [4–10]	< 0.001	5 [4–6]	5 [4–8]	ns	ns
Long-term outcomes*							
All-cause death, n (%)	117 (8.9)	143 (17.2)	< 0.001	15 (7.7)	8 (22.9)	0.006	ns
With T2DM	20 (16.9)	80 (18.4)	0.718	2 (15.4)	4 (26.7)	0.468	
Cardiovascular-death, n (%)	67 (5.1)	84 (10.1)	< 0.001	7 (3.6)	5 (14.3)	0.009	ns
With T2DM	11 (9.3)	51 (11.7)	0.46	1 (7.7)	3 (20.0)	0.35	
Re-AMI, n (%)	58 (4.4)	41 (4.7)	ns	1 (0.5)	0 (0)	ns	ns
Stroke, n (%)	2 (0.2)	1 (0.1)	ns	0 (0)	0 (0)	ns	ns
Heart failure, n (%)	108 (10.3)	103 (15.8)	0.001	8 (5.2)	4 (15.4)	ns	ns
MACE, n (%)	212 (16)	251 (28.6)	< 0.001	20 (10.3)	7 (18.4)	ns	ns
MAE, n (%)	260 (19.7)	304 (34.7)	< 0.001	27 (13.8)	10 (26.3)	ns	ns

Continuous variables are presented as median (IQR) while categorical ones as n (%)

*no-aHGL* admission normal glucose level, *aHGL* admission high glucose level, *T2DM* type 2 diabetes mellitus, *IABP* Intra-Aortic Balloon Pump, *AMI* acute myocardial infarction, *MACE* major adverse cardiovascular event, *MAE* major adverse event

Long term outcomes (*): MIOCA no-aHGL (N = 1308); MIOCA aHGL (N = 833); MINOCA no-aHGL (N = 194); MINOCA aHGL (N = 35)

**Fig. 1:**
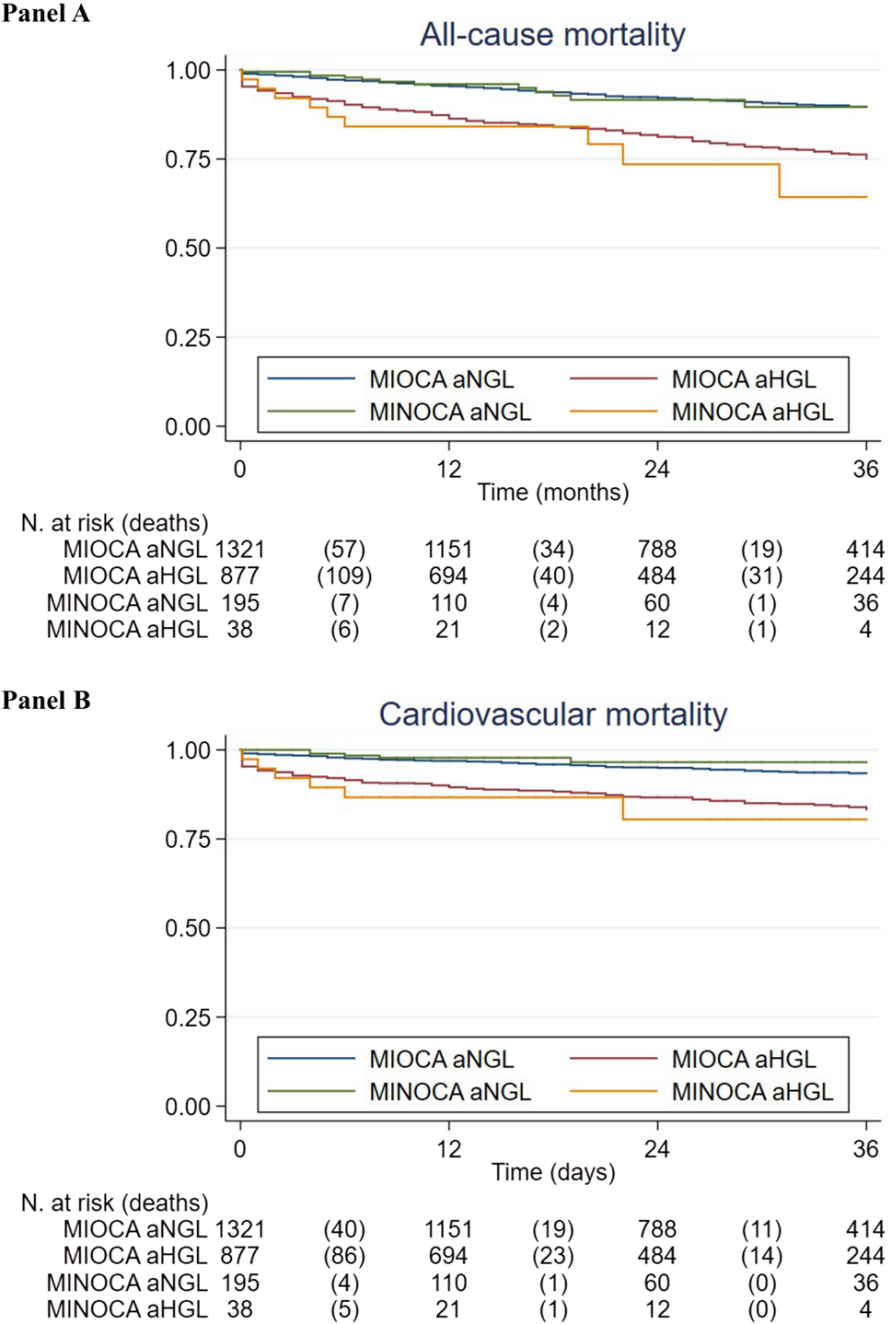
Kaplan–Meier survival curves in AMI patients with and without hyperglycemia. **A** All-cause mortality. Significant pairwise differences were found for MINOCA with and without hyperglycemia (p < 0.01), MIOCA with and without hyperglycemia (p < 0.001); MIOCA with hyperglycemia and MINOCA without hyperglycemia, (p < 0.05), MINOCA with hyperglycemia and MIOCA without hyperglycemia (p < 0.001), **B** cardiovascular mortality MIOCA with hyperglycemia and MINOCA without hyperglycemia (p = 0.011), MINOCA with hyperglycemia and MIOCA without hyperglycemia (p < 0.01), MINOCA with and without hyperglycemia (p = 0.0011), MIOCA with and without hyperglycemia (p < 0.001)

The median follow-up duration after discharge was 26 [14–38] months. Over this period, 283 deaths were recorded, 57.6% related to cardiovascular causes. In both MIOCA and MINOCA populations, all-cause mortality occurred more frequently among hyperglycemic patients (17.2% vs 8.9%, p < 0.001 and 22.9% vs 7.7%, p = 0.006, respectively). Similarly, cardiovascular deaths were more often observed in hyperglycemic patients, both in MIOCA and MINOCA cohorts (10.1% vs 5.1% p < 0.001 and 14.3% vs 3.6% p = 0.009, respectively) (Table 2). Kaplan–Meier estimates of patient survival at 3 years are shown in Fig. 1. Comparing MIOCA and MINOCA hyperglycemic patients, no differences in all-cause and cardiovascular mortality were observed (Fig. 1A, B). Accordingly, normoglycemic patients (both MIOCA and MINOCA) exhibited a similar long-term outcome (Fig. 1A, B). Macrovascular events (re-infarction and stroke) occurred almost exclusively in MIOCA patients, regardless of admission glucose levels. aHGL MINOCA patients exhibited a threefold incidence of heart failure episodes than normoglycemic, even though this result was not statistically significant. In contrast, hyperglycemic MIOCA cases showed a higher incidence of such occurrence than normoglycemic ones (15.8% vs 10.3%, p = 0.001) (Table 2).

In the multivariable Cox regression model, aHGL per se (HR = 4.221, 95% CI 1.867–9.499) or associated with T2DM (HR = 3.548, 95% CI 1.532–8.215) was a significant predictor of in-hospital mortality after adjusting for covariates (Table 3). As for the long-term outcome, both aHGL and T2DM were identified as independent predictors of all-cause mortality; notably, the combination of such conditions conferred a greater risk (HR = 1.87, 95% CI 1.35–2.59) than either condition alone (only aHGL: HR = 1.708, 95% CI 1.219–2.394; only T2DM: HR = 1.698, 95% CI 1.006–2.866) (Table 3). In the Cox regression model carried out in the overall sample, a 10 mg/dL increase of glucose level conferred a 4.6% increase in mortality risk, being 4.5% in MIOCA versus 9.9% in MINOCA patients. Finally, both for MIOCA and MINOCA, the mortality rate was similar between patients who were hyperglycemic both at admission and discharge compared to those who were no longer hyperglycemic at discharge.

**Table 3:** Cox regression analysis predicting short term all-cause mortality (a) and long term all-cause (b)

	HR	Std. err	p-value	95% CI
Short term all-cause mortality (a)				
Age, (1 year increase)	1.053	0.017	< 0.001	1.026–1.091
Female Gender	1.191	0.370	0.58	0.642–2.192
Hypertension	0.663	0.199	0.171	0.368–1.195
Smoking	0.540	0.179	0.063	0.282–1.033
Group				
No T2DM–no aHGL	Ref. cat			
Only aHGL	4.221	1.748	0.001	1.867–9.499
Only T2DM	–	–	–	–
aHGL + T2DM	3.548	1.520	0.003	1.532–8.215
Killip class > 1	2.711	0.904	0.003	1.410–5.210
MINOCA/MIOCA	0.437	0.358	0.304	0.046–2.614
Troponin I Peak IQR				
I	Ref cat			
II	0.367	0.262	0.160	0.090–1.486
III	1.544	0.770	0.384	0.581–4.105
IV	3.120	1.440	0.014	1.262–7.712
Long term all-cause mortality (b)				
Age (years)	1.093	0.009	< 0.001	1.076–1.110
Female Gender	0.891	0.133	0.441	0.665–1.194
Hypertension	1.600	0.315	0.017	1.088–2.354
Smoking habit	1.150	0.179	0.119	0.944–1.654
Group				
No T2DM–no aHGL	Ref. Cat			
Only aHGL	1.708	0.294	0.002	1.219–2.394
Only T2DM	1.698	0.454	0.047	1.006–2.866
aHGL + T2DM	1.870	0.310	< 0.001	1.351–2.588
Discharge LVEF	0.967	0.006	< 0.001	0.955–0.979
NSTEMI/STEMI	1.116	0.152	0.419	0.855–1.224
Left main	0.919	0.134	0.564	0.691–1.224
MINOCA/MIOCA	1.377	0.330	0.182	0.862–2.201

*no-aHGL* admission normal glucose level, *aHGL* admission high glucose level, *T2DM* type 2 diabetes mellitus, *LVEF* left ventricular ejection fraction, *NSTEMI* Non-ST-segment elevation myocardial infarction, *STEMI* ST-segment elevation myocardial infarction

## Discussion

This prospective observational study investigated the link between aHGL and AMI with a particular focus on prognostic information. Importantly, we evaluated for the first time the impact of hyperglycemia on MINOCA, a quite heterogeneous and still largely unexplored clinical entity. The main findings are: (i) aHGL is 2 times more frequent among MIOCA patients compared to MINOCA (37.6% vs 16.3%); (ii) aHGL carries a worse clinical profile in terms of baseline characteristics and hemodynamic instability; (iii) the expected clinical and prognostic impact of hyperglycemia on MIOCA patients was similarly observed in the context of MINOCA; (iv) aHGL accurately identified a group of high-risk patients for short-term outcomes; (v) the prognostic role of hyperglycemia is maintained over time resulting in an adverse long-term outcome, conferring an adjunctive risk to the sole diabetic condition; (vi) after AMI, the prognosis is strongly influenced by the gluco-metabolic status independently of the coronary anatomy.

### Hyperglycemia and short-term outcomes

The incidence of aHGL in the context of AMI ranges from 20 to 50% [26], depending on the definition of stress hyperglycemia, which varies from 140 to 180 mg/dL. By setting the cut-off at 140 mg/dL as proposed by the American Heart Association Diabetes Committee [2], we found an overall prevalence of 37.6%, ranging from 40% in MIOCA to 16.3% in MINOCA. The latter data is particularly relevant as aHGL was estimated for the first time in a systematically studied MINOCA population. Indeed, such a difference in the glycemic status should not be surprising given that MINOCA subjects are usually females with a lower atherosclerotic burden as expressed by fewer cardiovascular risk factors [16]. Nevertheless, our study revealed that stress hyperglycemia was homogeneously associated with a worse functional status in all AMI patients, including those falling into the current definition of MINOCA. In particular, hyperglycemic patients were older, overweight, with cardiovascular risk factors and comorbidities, both in MIOCA and MINOCA groups.

Moreover, our results showed that even the clinical conditions at hospital admission in hyperglycemic patients were overall characterized by signs of hemodynamic instability and heart failure, with a higher heart rate and Killip class and a greater prevalence of atrial fibrillation. All these clinical and instrumental indices are paralleled by a higher GRACE score observed in hyperglycemic subjects, both in MIOCA and MINOCA groups. Consequently, it was intriguing to assess the interplay between aHGL and short- and long-term prognosis. Previous studies have demonstrated a hyperglycemia-related mortality risk in AMI patients, both diabetic and non-diabetic, without however distinguishing between MIOCA and MINOCA [1]. Notably, our data corroborate and support the current knowledge, adding however a crucial piece to the puzzle: hyperglycemia maintains its prognostic relevance independently of coronary stenoses.

From a pathophysiological point of view, in the early stage of AMI, hyperglycemia promotes a prothrombotic state, increases inflammation and sympathetic nervous system activity, worsens endothelium function, and imbalances the oxidative stress releasing reactive oxygen species [21, 27, 28]. As a result, all these changes impair coronary microvascular function with an increased risk of no-reflow phenomenon [29, 30]. Reasonably, this evidence derives mainly from hyperglycemic MIOCA cohorts that exhibit a larger infarct size, potentially explaining the adverse events occurring during hospitalization [31]. Similarly, in the context of MINOCA, a “hyperglycemic environment” can impact both macro and micro-circulation, producing endothelial erosion and plaque disruption, epicardial and microvascular coronary spasm, coronary thrombosis, and microvascular dysfunction, overall intensifying the underlying pathophysiologic mechanisms. In our study, MINOCA patients presented a trivial infarct size, and only 2 in-hospital deaths were recorded. Interestingly, a high incidence of intra-hospital arrhythmias was observed among hyperglycemic patients, both in MIOCA and MINOCA. Plausible mechanisms for this occurrence may be related to insulin resistance and catecholamine overproduction, leading to lipolysis and the release of circulating free fatty acids. The latter induces two potential toxic effects on the ischemic myocardium: damage of cardiac-cell membranes and calcium overload, consequently increasing the arrhythmic burden and reducing myocardial contractility [32]. Notably, in our overall study population, aHGL emerged as the strongest independent predictive factor of short-term mortality, highlighting the utility of such a quick and accessible parameter to identify high-risk patients.

### Hyperglycemia and long-term outcomes

In terms of long-term prognosis, aHGL patients—both in MIOCA and MIOCA groups—exhibited a higher rate of all-cause and cardiovascular mortality. The prognostic role of aHGL appears to be unrelated to secondary prevention medical therapy. Specifically, our MIOCA patients were quite homogenously treated in terms of discharge medical therapy with the only exception of beta-blockers which were more often administrated in hyperglycemics. Indubitably our results confirm the existing that MINOCA is still an “undertreated population” with no differences in relation to admission glucose levels, except for antithrombotic therapies as hyperglycemic patients more often required oral anticoagulants.

Macrovascular complications (stroke and re-AMI) were recorded in MIOCA more often than in MINOCA patients, reflecting the high burden of atherosclerosis in obstructive ischemic disease. Furthermore, hyperglycemic patients experienced over time more hospitalization for heart failure within the MIOCA cohort, while a trend was observed in MINOCA. These results might ultimately confirm recent data which demonstrated that microvascular dysfunction plays a role in the pathophysiological mechanism of heart failure with preserved ejection fraction [33–35]. This concept is particularly relevant and underlies useful clinical implications: the approach to AMI should always go beyond coronary stenoses as MINOCA patients might still have an eventful prognosis, potentially characterized either by the recurrence of acute coronary syndromes or by stable angina and episodes of heart failure with preserved ejection fraction. In fact, all these clinical entities supposedly represent different sides of the same “pathophysiological coin”, which is functional coronary dysfunction.

Another important aspect of our work was the search for risk factors predicting long-term outcomes. Our analysis showed that both hyperglycemia and T2DM per se have a long-lasting prognostic impact after AMI, regardless of the anatomic substrate—MIOCA or MINOCA. Notably, combining the two conditions seems to confer an adjunctive risk, highlighting the importance of optimal gluco-metabolic control. Although the prognostic role of diabetes is well known, we assessed the long-lasting impact of a simple parameter such as aHGL on all patients admitted for AMI, including for the first time those fulfilling the current diagnostic criteria of MINOCA. Indeed, both diabetes and hyperglycemia directly influence atherosclerotic plaque formation and progression and may induce microvascular dysfunction and microangiopathy. Therefore, the resulting vicious circle might affect the macrovascular and microvascular beds, leading to an adverse long-term prognosis in both MIOCA and MINOCA patients [36–38].

**Fig. 2:**
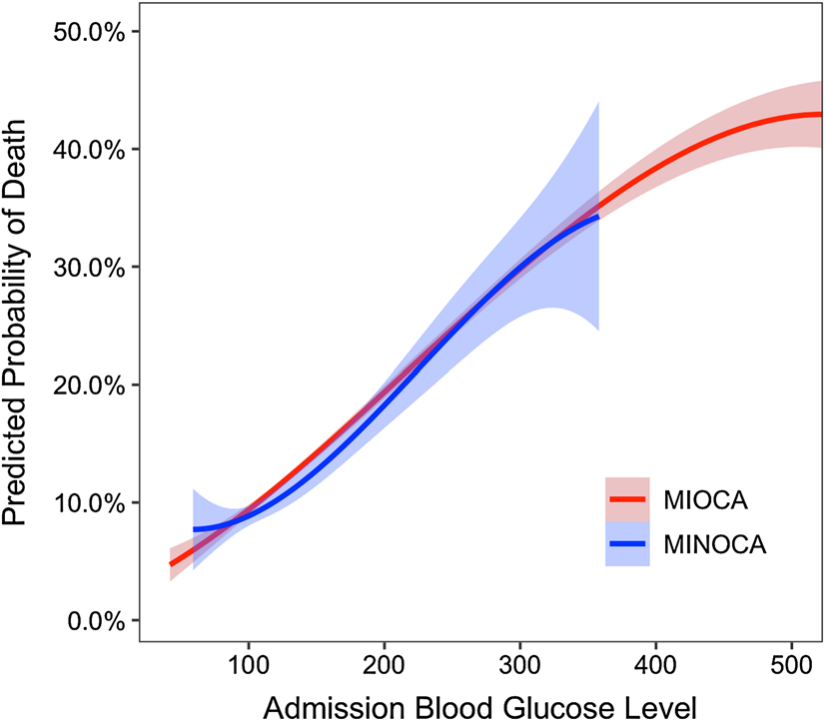
Predicted probability of all-cause death per groups according to admission blood glucose levels presented as continuous variable. MIOCA: red curve; MINOCA: blue curve

Thus, the pivotal issue of our study is that we proved for the first time that in MINOCA patients, a simple measurement of blood glucose levels at hospital admission could impact both short- and long-term prognosis. Consequently, in the setting of AMI, the prognosis is strongly influenced by the gluco-metabolic status independently of the underlying coronary anatomy (Fig. 2). Indeed, in the heterogeneous world of MINOCA, a quick and widely accessible parameter such as aHGL can accurately identify a group of “high-risk” patients who could probably benefit from a proper secondary prevention medical therapy.

### Study limitations

Our results should be interpreted in light of some limitations. First of all, analyses were conducted on relatively small sample size, especially regarding the MINOCA cohort, even though all these patients were well characterized aiming to exclude other causes of acute myocardial injury. Therefore, our findings should be interpreted as exploratory and larger studies are needed to confirm the prognostic impact of hyperglycemia in MINOCA patients. Secondly, aHGL levels may have been influenced by multiple factors such as last meal composition and timing and day versus night measurements. Moreover, in patients with suspected DM, no definite rule-out criteria were adopted, therefore some diabetes diagnoses may have been missed. However, it should be noted that not all patients admitted for AMI can undergo an oral glucose tolerance test, especially in the acute phase. Lastly, our follow-up data did not include laboratory information regarding the gluco-metabolic status.

## Conclusions

Admission hyperglycemia, regardless of a concomitant DM diagnosis, is a simple and useful predictor of short and long-term outcomes in patients with AMI, both with obstructive coronary artery disease and MINOCA. Nevertheless, in all patients hospitalized for AMI, the combination of hyperglycemia and DM seems to further negatively impact prognosis, highlighting the importance of an optimal glucometabolic control. Since aHGL is promptly available in all AMI cases, this biomarker may be incorporated into risk calculation models to identify high-risk patients for early and late mortality. This rapid stratification is potentially particularly useful in the heterogeneous context of MINOCA, where secondary prevention strategies still lack standardization. Larger multicentric studies are required to validate our findings and fully unravel the complex interplay between hyperglycemia and ischemic heart disease.

## Supplementary Information

**Additional file 1. **Supplementary File 1

## Data Availability

The datasets used and/or analysed during the current study are available from the corresponding author on reasonable request.

## Abbreviations

T2DM: Type 2 diabetes mellitus
AMI: Acute myocardial infarction
MINOCA: Myocardial infarction with non-obstructive coronary arteries
MACE: Major adverse cardiovascular events
aHGL: Admission high glucose levels
MIOCA: Myocardial infarction with obstructive coronary arteries
CAG: Coronary angiography
STEMI: ST-segment elevation acute myocardial infarction
NSTEMI: Non-ST segment elevation acute myocardial infarction
LVEDV: Left-ventricular-end-diastolic-volume
LVEF: Left ventricular ejection fraction
