# Supplementary material for: Impact of admission hyperglycemia on short and long-term prognosis in acute myocardial infarction: MINOCA versus MIOCA
Source: Cardiovasc Diabetol. 2021 Sep 24;20:192. doi: 10.1186/s12933-021-01384-6 (PMC8464114; doi:10.1186/s12933-021-01384-6)
Supplement: Supplementary file 1 — Additional file 1. Supplementary File 1 [file 12933_2021_1384_MOESM1_ESM.docx]

**Online-Only Additional file**

**Impact of Admission Hyperglycemia on Short and Long-Term Prognosis in Acute Myocardial Infarction: MINOCA versus MIOCA.**

**METHODS**

**Data collection**

For each patient, demographic and baseline clinical data were collected, including age, sex, anthropometric data, cardiovascular risk factors, family history of cardiovascular disease, history of comorbidities, first admission diagnosis and discharge therapy. A standard 12-lead EKG, transthoracic echocardiography following the latest European recommendations^1^ and laboratory tests were performed in all cases. The glomerular filtration rate was calculated using the CKD-EPI formula. We also collected information on major epicardial coronary arteries obstruction based on visual assessment during acute CAG performed by an expert interventional cardiologist.

**Definition of hyperglycemia and diabetes**

Admission blood glucose level were determined at the time of hospital admission as part of the standard evaluation by the first venous blood samples routinely drawn at the Emergency Room or Intensive Care Unit and analyzed at the central laboratory of the hospital. Stress hyperglycemia was defined as plasma glucose levels greater than 140 mg/dL at any given time during hospitalization in diabetic and non-diabetic patients^2,3^. According to the admission glucose level, patients were classified as non-hyperglycemic < 140 mg/dl and hyperglycemic ≥ 140 mg/dl. Hyperglycemic subjects with admission glucose level > 200 mg/dl received insulin–glucose infusion to decrease blood glucose between 126 and 199 mg/dl for the first 24 hours, followed by subcutaneous insulin injections^4,5^. Pre-existing DM was defined as a known reported history of DM at admission, either treated with diet and lifestyle measures alone or with the additional use of oral glucose-lowering medications and insulin. Diagnosis of DM was made during hospitalization based on fasting plasma glucose levels ≥ 126mg/dL or glycosylated haemoglobin (HbA1c) ≥ 48 mmol/mol^6^. Patients without a history of DM and with HbA1c < 48 mmol/mol were considered non-diabetic.

**Clinical Outcomes**

Pre-specified endpoints were all-cause mortality, cardiovascular deaths, arrhythmias, myocardial infarction, heart failure, ischemic stroke, and MACE. All-cause mortality included all causes of death for the population during the follow-up^7^. Cardiovascular mortality consisted of deaths that result from an AMI, sudden cardiac death, heart failure, stroke, and other cardiovascular causes^7^. Heart failure, both with preserved and reduced ejection fraction, was diagnosed according to current ESC guidelines^8^. MAE comprises all-cause mortality, non-fatal re-AMI, non-fatal heart failure, and non-fatal ischemic stroke. MACE is a composite of cardiovascular mortality, non-fatal re-AMI, non-fatal heart failure, and non-fatal ischemic stroke.

**Sample size calculation**

Sample size estimation was calculated by considering as the main outcomes the long-term all-cause death. We calculated a sample size of 35 hyperglycemic MINOCA patients, with estimated 90% power to detect a change of 0.01 between the study endpoints of the hyperglycemics and normoglycemics groups at 5% level of significance. A 10% loss due to early withdrawals and non-evaluable measurements was assumed and combined with the effect of stratification on analysis, resulted in the requirement to recruit at least 35 patients per group.

**References**

1. Lang RM, Badano LP, Mor-Avi V, Afilalo J, Armstrong A, Ernande L, Flachskampf FA, Foster E, Goldstein SA, Kuznetsova T, Lancellotti P, Muraru D, Picard MH, Rietzschel ER, Rudski L, Spencer KT, Tsang W, Voigt J-U. Recommendations for Cardiac Chamber Quantification by Echocardiography in Adults: An Update from the American Society of Echocardiography and the European Association of Cardiovascular Imaging. *Eur Heart J – Cardiovasc Imaging* 2015;16:233–271.
2. Deedwania P, Kosiborod M, Barrett E, Ceriello A, Isley W, Mazzone T, Raskin P. Hyperglycemia and Acute Coronary Syndrome: A Scientific Statement From the American Heart Association Diabetes Committee of the Council on Nutrition, Physical Activity, and Metabolism. *Circulation* 2008;117:1610–1619.
3. Umpierrez GE, Hellman R, Korytkowski MT, Kosiborod M, Maynard GA, Montori VM, Seley JJ, Van den Berghe G. Management of Hyperglycemia in Hospitalized Patients in Non-Critical Care Setting: An Endocrine Society Clinical Practice Guideline. *J Clin Endocrinol Metab* 2012;97:16–38.
4. Malmberg K, Rydén L, Efendic S, Herlitz J, Nicol P, Waldenstrom A, Wedel H, Welin L. Randomized trial of insulin-glucose infusion followed by subcutaneous insulin treatment in diabetic patients with acute myocardial infarction (DIGAMI study): Effects on mortality at 1 year. *J Am Coll Cardiol* 1995;26:57–65.
5. Malmberg K, Rydén L, Wedel H, Birkeland K, Bootsma A, Dickstein K, Efendic S, Fisher M, Hamsten A, Herlitz J, Hildebrandt P, MacLeod K, Laakso M, Torp-Pedersen C, Waldenström A; DIGAMI 2 Investigators. Intense metabolic control by means of insulin in patients with diabetes mellitus and acute myocardial infarction (DIGAMI 2): effects on mortality and morbidity. *Eur Heart J.* 2005 Apr;26(7):650-61.
6. American Diabetes Association. 6. Glycemic Targets: *Standards of Medical Care in Diabetes—2021*. *Diabetes Care* 2021;44:S73–S84.
7. Hicks KA, Mahaffey KW, Mehran R, Nissen SE, Wiviott SD, Dunn B, Solomon SD, Marler JR, Teerlink JR, Farb A, Morrow DA, Targum SL, Sila CA, Hai MTT, Jaff MR, Joffe HV, Cutlip DE, Desai AS, Lewis EF, Gibson CM, Landray MJ, Lincoff AM, White CJ, Brooks SS, Rosenfield K, Domanski MJ, Lansky AJ, McMurray JJV, Tcheng JE, Steinhubl SR, et al. 2017 Cardiovascular and Stroke Endpoint Definitions for Clinical Trials. *Circulation* 2018;137:961–972.
8. Ponikowski P, Voors AA, Anker SD, Bueno H, Cleland JG, Coats AJ, Falk V, González-Juanatey JR, Harjola VP, Jankowska EA, Jessup M, Linde C, Nihoyannopoulos P, Parissis JT, Pieske B, Riley JP, Rosano GM, Ruilope LM, Ruschitzka F, Rutten FH, van der Meer P; Authors/Task Force Members; Document Reviewers. 2016 ESC Guidelines for the diagnosis and treatment of acute and chronic heart failure: The Task Force for the diagnosis and treatment of acute and chronic heart failure of the European Society of Cardiology (ESC). Developed with the special contribution of the Heart Failure Association (HFA) of the ESC. Eur J Heart Fail. 2016 Aug;18(8):891-975.

**Figure Legend**

**Figure S1: flow chart Study**. CAG: coronary angiography; AMI: acute myocardial infarction; TpNOCA: troponin-positive non-obstructive coronary arteries; MIOCA: obstructive myocardial infarction; MINOCA: myocardial infarction with non-obstructive coronary arteries; aBGL: admission blood glucose level; aHGL: admission high glucose level; no-aHGL: admission normal glucose level.

**Figure S2: frequency distribution of glycemia with a cut-off at 140 mg/dl for both groups**. Panel A: MIOCA patients; Panel B: MINOCA patients.

**Table S1: angiographic result of MIOCA and MINOCA patients**

|  | **MIOCA N = 2198** | | **MINOCA N = 233** | | |
| --- | --- | --- | --- | --- | --- |
|  | **STEMI N = 987** | **NSTEMI N = 1211** |  | **STEMI  N = 27** | **NSTEMI N = 206** |
| **Target Lesion** |  |  | **Causes** |  |  |
| LM lesion, n (%) | 41 (4.1) | 162 (13.3) | Epicardial coronary spasm | 1 (3.7) | 13 (6.3) |
| LAD lesion, n (%) | 807 (81.7) | 926 (76.4) | SCAD | 8 (29.6) | 25 (12.1) |
| LCx lesion, n (%) | 384 (38.9) | 481 (39.7) | Coronary embolism | 4 (14.8) | 0 (0) |
| RC lesion, n (%) | 651 (65.9) | 714 (58.9) | Atherosclerotic plaque disruption (type I) | 5 (18.5) | 30 (14.6) |
|  |  |  | Supply-demand mismatch (type II) | 9 (33.3) | 138 (67) |
| **Number of Vessels** |  | | **Number of vessels with stenosis** |  |  |
| LM, n (%) | 31 (3.1) | 133 (10.9) | LM (1-20%), n (%) | 4 (14.8) | 1 (0.5) |
| 1 Vessel, n (%) | 611 (61.9) | 406 (33.5) | 1 Vessel (20-49%), n (%) | 3 (11.1) | 31 (15) |
| 2 Vessels, n (%) | 264 (26.7) | 345 (28.5) | 2 Vessels (20-49%), n (%) | 3 (11.1) | 12 (5.8) |
| 3 Vessels, n (%) | 81 (8.2) | 327 (27) | 3 Vessels (20-49%), n (%) | 0 (0) | 3 (1.4) |

AMI: Acute Myocardial Infarction; LAD: Left Anterior Descending artery; LCx: Left Circumflex; LM: Left Main; MINOCA: myocardial Infarction with Non-Obstructive Coronary Arteries; NSTEMI: Non-ST-segment elevation myocardial infarction; RC: Right Coronary artery; STEMI: ST-segment elevation myocardial infarction; SCAD: spontaneous coronary artery dissection.

**Table S2: admission, intra-hospital and discharge medical therapy of MIOCA and MINOCA ACS patients, according to admission hyperglycemia.**

|  | MIOCA  N = 2198 | |  | | MINOCA  N = 233 | |  | HGL MIOCA vs MINOCA |
| --- | --- | --- | --- | --- | --- | --- | --- | --- |
|  | **no-aHGL**  **N = 1321** | **aHGL**  **N = 877** | **p-value** | **no-aHGL**  **N = 195** | | **aHGL**  **N = 38** | **p-value** | **p-value** |
| Admission Medical Therapy |  |  |  |  | |  |  |  |
| Aspirin, n (%) | 461 (35) | 342 (39) | 0.05 | 50 (25.6) | | 8 (21.1) | ns | 0.026 |
| P2Y12 Inhibitor, n (%) | 110 (8.3) | 97 (11) | 0.04 | 11 (5.6) | | 2 (5.3) | ns | ns |
| DAPT, n (%) | 59 (4.5) | 42 (4.8) | ns | 2 (1) | | 1 (2.6) | ns | ns |
| Beta-blockers, n (%) | 478 (36.5) | 362 (41.7) | 0.016 | 58 (29.7) | | 17 (44.7) | ns | ns |
| RAAS inhibitors, n (%) | 605 (46.1) | 459 (53) | 0.002 | 63 (32.3) | | 22 (58) | 0.003 | ns |
| Statins, n (%) | 356 (27.2) | 266 (30.6) | ns | 50 (25.6) | | 14 (36.8) | ns | ns |
| VKA, n (%) | 53 (4) | 53 (6.1) | 0.029 | 9 (4.6) | | 6 (15.8) | 0.011 | 0.018 |
| NAO, n (%) | 39 (3) | 20 (2.3) | ns | 9 (4.6) | | 6 (15.8) | 0.011 | 0.001 |
| Thiazide diuretics, n (%) | 154 (11.8) | 105 (12.1) | ns | 17 (8.7) | | 5 (13.2) | ns | ns |
|  |  |  |  |  | |  |  |  |
| Admission Glucose-lowering agents |  |  |  |  | |  |  |  |
| Insulin sensitizers (metformin), n (%) | 60 (4.6) | 238 (27.3) | < 0.001 | 8 (4.1) | | 5 (15.6) | 0.009 | ns |
| Insulin providers (sulfonylureas), n (%) | 32 (2.4) | 151 (17.3) | < 0.001 | 2 (1) | | 3 (9.4) | 0.003 | ns |
| DPP-4 Inhibitors, n (%) | 7 (0.5) | 26 (3) | < 0.001 | 1 (0.5) | | 1 (3.1) | ns | ns |
| GLP-1 Agonist, n (%) | 2 (0.2) | 7 (0.8) | 0.02 | / | | / | / | ns |
| SGLT-2 Inhibitors, n (%) | 1 (0.1) | 3 (0.3) | ns | / | | / | / | ns |
| Insulin, n (%) | 23 (1.7) | 108 (12.4) | < 0.001 | 0 | | 2 (6.3) | / | ns |
|  |  |  |  |  | |  |  |  |
| First 48H glucose-lowering strategy |  |  |  |  | |  |  |  |
| E.v. Insulin, n (%) | 0 (0) | 79 (9.1) | < 0.001 | 0 (0) | | 0 (0) | / | ns |
| DTx, n (%) | 85 (6.5) | 422 (48.5) | < 0.001 | 7 (3.7) | | 6 (22.2) | < 0.001 | 0.007 |
|  |  |  |  |  | |  |  |  |
| Discharge Medical Therapy |  |  |  |  | |  |  |  |
| Aspirin, n (%) | 1249 (96.4) | 787 (95.3) | ns | 155 (81.2) | | 22 (59.5) | 0.004 | < 0.001 |
| P2Y12 Inhibitor, n (%) | 1186 (91.5) | 758 (91.8) | ns | 110 (57.6) | | 16 (43.2) | ns | < 0.001 |
| DAPT, n (%) | 1167 (90) | 737 (89.2) | ns | 103 (54) | | 10 (27) | 0.003 | < 0.001 |
| Beta-blockers, n (%) | 1109 (85.7) | 734 (89) | 0.029 | 142 (74.3) | | 29 (78.4) | ns | 0.048 |
| RAAS inhibitors, n (%) | 1023 (79) | 644 (78) | ns | 126 (66) | | 27 (73) | ns | ns |
| Statins, n (%) | 1167 (90.2) | 730 (88.4) | ns | 139 (72.8) | | 26 (70.3) | ns | 0.001 |
| VKA, n (%) | 85 (6.6) | 92 (11.1) | < 0.001 | 15 (7.9) | | 5 (13.5) | ns | ns |
| NOAC, n (%) | 45 (3.5) | 37 (4.5) | ns | 18 (9.5) | | 8 (21.6) | 0.034 | < 0.001 |
|  |  |  |  |  | |  |  |  |
| Discharge Glucose-lowering agents |  |  |  |  | |  |  |  |
| Insulin sensitizers (metformin), n (%) | 57 (4.4) | 219 (30.6) | < 0.001 | 8 (4.2) | | 5 (20.8) | 0.01 | ns |
| Insulin providers (sulfonylureas), n (%) | 24 (1.9) | 126 (17.6) | < 0.001 | 2 (1.1) | | 3 (12.5) | < 0.001 | ns |
| DPP-4 Inhibitors, n (%) | 6 (0.5) | 31 (4.3) | < 0.001 | 1 (0.5) | | / | / | ns |
| GLP-1 Agonist, n (%) | 2 (0.2) | 6 (0.8) | 0.019 | / | | / | / | ns |
| SGLT-2 Inhibitors, n (%) | 1 (0.1) | 8 (1.1) | 0.001 | / | | / | / | ns |
| Insulin, n (%) | 22 (1.7) | 157 (22) | < 0.001 | 2 (1.1) | | 2 (8.3) | 0.013 | ns |

Continuous variables are presented as median (IQR) while categorical ones as n (%). Abbreviations: no-aHGL = admission normal glucose level; aHGL = admission high glucose level; RAAS = Renin-angiotensin-aldosterone system; VKA = Vitamin K antagonists; NOAC = Novel oral anticoagulants; DPP-4 = dipeptidyl peptidase 4; GLP-1 = glucagon-like peptide 1; SGLT-2 = sodium glucose co-transporter 2.

**Figure S1**

**
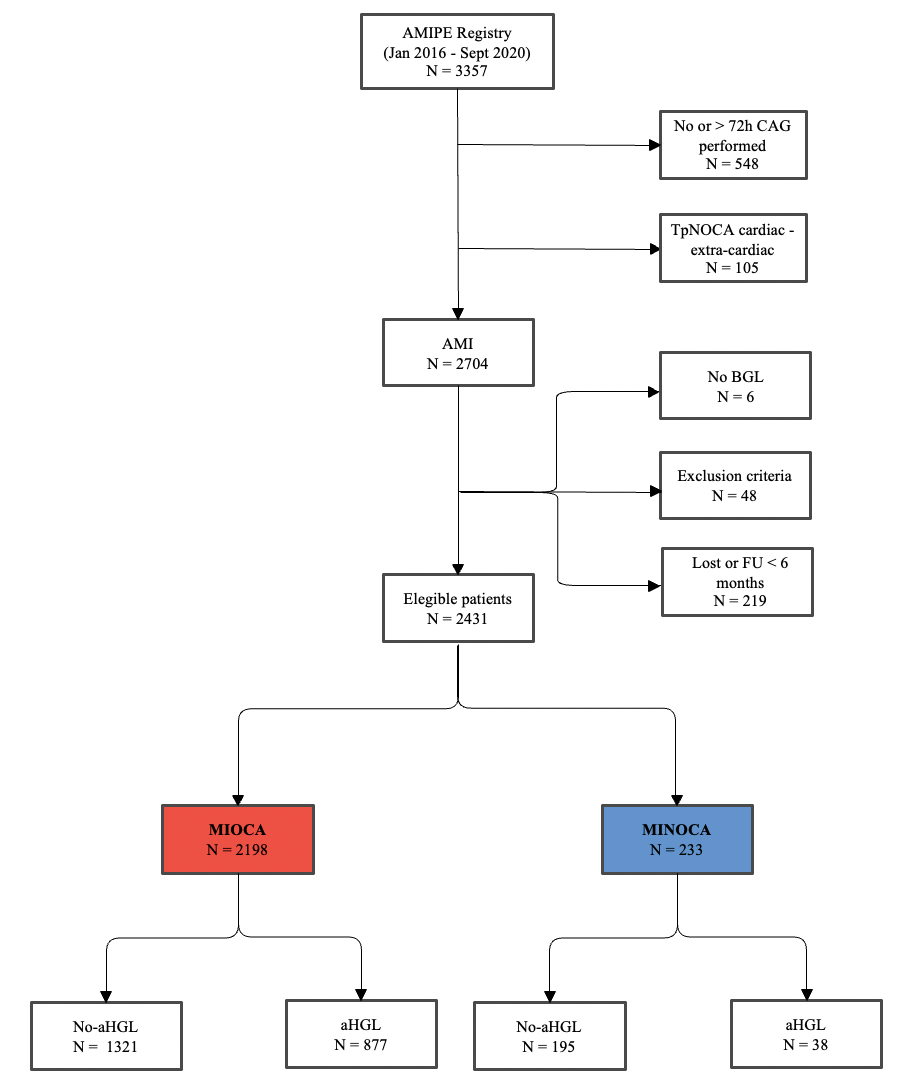
**

**Figure S2**

**
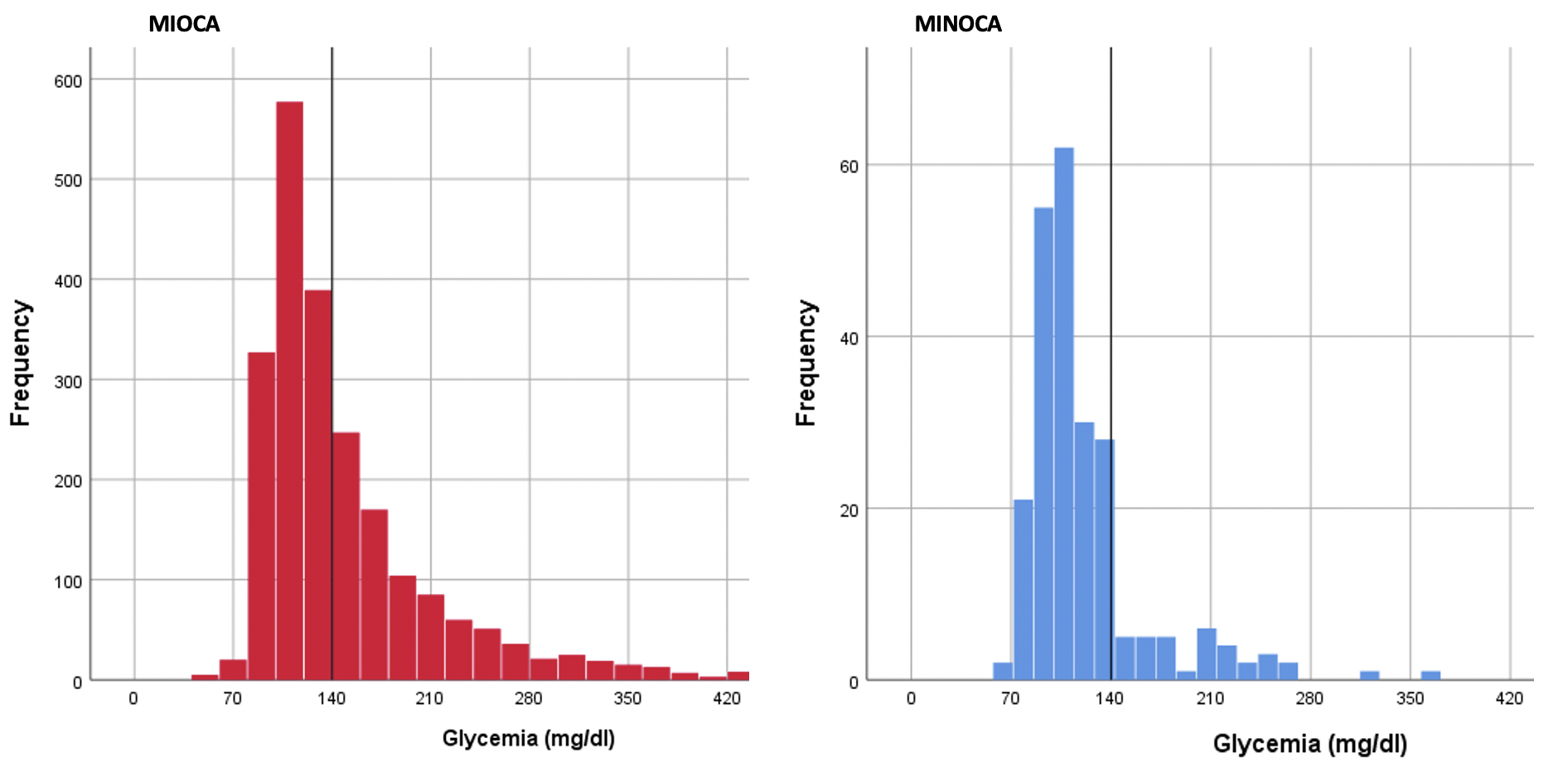
**
